# A proteomic analysis of serum-derived exosomes in rheumatoid arthritis

**DOI:** 10.1186/s41927-018-0041-8

**Published:** 2018-11-27

**Authors:** Hirotaka Tsuno, Mitsumi Arito, Naoya Suematsu, Toshiyuki Sato, Atsushi Hashimoto, Toshihiro Matsui, Kazuki Omoteyama, Masaaki Sato, Kazuki Okamoto, Shigeto Tohma, Manae S. Kurokawa, Tomohiro Kato

**Affiliations:** 1 Clinical Proteomics and Molecular Medicine, St. Marianna University Graduate School of Medicine, 2-16-1, Sugao, Miyamae, Kawasaki, Kanagawa 216-8511 Japan; 2 Department of Rheumatology, National Hospital Organization Sagamihara National Hospital, 18-1, Sakuradai, Minami, Sagamihara, Kanagawa 252-0392 Japan; 3 National Hospital Organization Tokyo National Hospital, 3-1-1, Takeoka, Kiyose, Tokyo, 204-8585 Japan; 4 Disease Biomarker Analysis and Molecular Regulation, St. Marianna University Graduate School of Medicine, 2-16-1, Sugao, Miyamae, Kawasaki, Kanagawa 216-8511 Japan; 5 The Institute of Scientific and Industrial Research, Osaka University, 8-1, Mihogaoka, Osaka, Ibaraki 567-0047 Japan

**Keywords:** Exosome, Proteomics, Rheumatoid arthritis, Osteoarthritis, Toll like receptor 3

## Abstract

**Background:**

To understand the roles of serum exosomes in rheumatoid arthritis (RA), we comprehensively investigated the protein profiles of serum exosomes in patients with RA.

**Methods:**

Exosomes were isolated from serum samples obtained from 33 patients (12 with active RA [aRA], 11 with inactive RA [iRA], 10 with osteoarthritis [OA]) and 10 healthy donors (HLs). Proteins extracted from the exosomes were separated by two-dimensional differential gel electrophoresis (2D-DIGE) and identified by mass spectrometry.

**Results:**

In total, 204 protein spots were detected by 2D-DIGE. In the aRA, iRA, and OA groups, 24, 5, and 7 spots showed approximately ≥ ±1.3-fold intensity differences compared with the HL group, respectively. We were able to identify proteins in six protein spots. Among them, the protein spot identified as Toll-like receptor 3 (TLR3) showed approximately 6-fold higher intensity in the aRA group than in the other groups.

**Conclusions:**

Patients with active RA possessed considerably different protein profiles of serum exosomes from patients with iRA, patients with OA, and healthy donors. The unique protein profile of serum exosomes, such as the possession of abundant TLR3 fragments, may reflect the pathophysiology of active RA.

**Electronic supplementary material:**

The online version of this article (10.1186/s41927-018-0041-8) contains supplementary material, which is available to authorized users.

## Background

Exosomes are nanometer-sized (50–100 nm) membranous extracellular vesicles secreted from various types of cells [1]. Exosomes are initially generated by inward budding of late endosomes, called “multivesicular bodies (MVBs)”, in cells and then released from the cells by the fusion of MVBs with plasma membranes [1]. Exosomes are involved in intercellular communications via the delivery of proteins, lipids, and RNAs (mRNA and microRNA) through exosome-cell fusion and via signal delivery through exosome-cell interactions [2, 3].

Immunologically, exosomes play roles in the presentation of antigens to T cells [4]. Exosomes derived from antigen-presenting cells (APCs) were reported to possess major histocompatibility complex (MHC) class I and II molecules, co-stimulatory molecules of CD80 and CD86, and adhesion molecule of intercellular adhesion molecule (ICAM)-1, which are essential to antigen presentation [5, 6]. In fact, exosomes derived from dendritic cells (DCs) have been shown to induce CD8+ T cells to produce interferon (IFN)-γ and tumor necrosis factor (TNF)-α in an exosomal MHC class I-dependent manner [7]. Furthermore, mature DC-derived exosomes have been reported to induce the proliferation of CD4+ T cells in an antigen-specific manner [8].

In the field of cancer, the immunosuppressive and immunostimulatory properties of exosomes have been demonstrated [4]. For example, tumor-derived exosomes were reported to induce T cell apoptosis via Fas ligand [9] and to inhibit interleukin (IL)-2-induced proliferation of T cells [10]. Quite recently, exosomal programmed death-ligand 1 (PD-L1) was shown to inhibit the function of CD8+ T cells and facilitate the progression of melanoma [11].

Collectively, exosomes are thought to play immuno-pathological roles in various diseases. Rheumatoid arthritis (RA) is a systemic autoimmune disorder characterized by inflammation and proliferation of synovial tissue, destruction of cartilage and bone, and production of autoantibodies, such as anti-citrullinated peptide antibodies [12]. Although the pathogenesis of RA remains unclear, the pathophysiology is thought to be as follows: First, innate immunity activates DCs [13]. Subsequently, DCs migrate into central lymphoid organs and present autoantigens, such as type II collagen and citrullinated proteins, to T cells [13]. The activated T cells then induce B cells to produce autoantibodies [14]. These lymphocytes migrate into synovial tissue and enhance adaptive immune responses, leading to synovial angiogenesis and hyperplasia of synovial tissue [14]. Inflammatory cytokines such as IL-1, IL-6, IL-17, and TNF-α produced in this process amplify osteoclast differentiation and activation, which results in the destruction of cartilage and bone [12].

As mentioned above, exosomes have various immunological functions and are thus expected to play roles in the pathophysiology of RA. For instance, fibroblast-like synoviocyte (FLS)-derived exosomes were reported to contain citrullinated proteins [15] and a membrane form of TNF-α [16]. IL-1β-stimulated FLS-derived exosomes were reported to up-regulate the matrix metalloproteinase (MMP)-13 expression in chondrocytes [17]. We showed that IL-1β and anti-rheumatic drugs of salazosulfapyridine (SASP) and methotrexate (MTX) considerably altered the protein profiles of exosomes derived from SW982 of synovial sarcoma cell line [18]. Recently, it was reported that some serum exosomal proteins in patients with RA might be useful parameters of disease activity [19]. In terms of bone formation and resorption, osteoclast-derived exosomes containing microRNA (miR-214) were reported to play an inhibitory role in osteoblast activity [20, 21]. However, at present, the roles of exosomes in RA are largely unclear.

These previous findings suggest that characterizing the exosomes generated in patients with RA would be quite useful. We therefore characterized the protein profile of serum exosomes in patients with RA by two-dimensional differential gel electrophoresis (2D-DIGE) and a subsequent mass spectrometry (MS) analysis. We found that the serum exosomes in patients with active RA (aRA) possessed considerably different protein profiles from those in patients with inactive RA (iRA), patients with osteoarthritis (OA), and healthy donors (HLs). In particular, the serum exosomes in patients with aRA contained abundant Toll-like receptor (TLR) 3 fragments. Our data will help promote the understanding of the roles of exosomes in the pathophysiology of RA.

## Materials and methods

### Preparation of blood samples

Blood samples were obtained from 33 patients (12 patients with aRA, 11 with iRA, 10 with OA) and 10 HLs. Clinical information on the patients with RA is shown in Table 1. The diagnoses of RA and OA were made according to the respective criteria by American College of Rheumatology [22, 23]. We defined aRA as “Disease Activity Score 28 using C-reactive protein (DAS28-CRP) > 2.7” and iRA as “DAS28-CRP < 2.3” [24]. Blood samples were drawn into vacuum tubes (Venoject II, VPAS109K50; Terumo Corporation, Tokyo, Japan) and centrifuged at 1500 *g* for 10 min to separate the sera at room temperature. The separated serum samples were then stored at − 80 °C until use.

**Table 1:** Clinical information of patients with aRA, iRA, and OA and of HL

	Active RA (*n* = 12)	Inactive RA (*n* = 11)	*p* Value (aRA vs. iRA)	OA (*n* = 10)	HL (*n* = 10)
Age (years), mean (range)	62.2 (31 to 79)	63.4 (41 to 80)	0.85	62.5 (41 to 74)	60.5 (41 to 82)
Sex, male/female	2/10	3/8	0.93	1/9	2/8
Disease duration (years), mean (range)	5.6 (0.25 to 20)	10.9 (0.25 to 38)	0.22	(−)	(−)
ESR (mm/h), mean (range)	56.3 (16 to 100)	21.0 (2 to 41)	< 0.01	(−)	(−)
CRP (mg/L), mean (range)	3.19 (0.07 to 7.16)	0.14 (0.02 to 0.33)	< 0.01	(−)	(−)
MMP-3 (ng/mL), mean (range)	252.0 (18.8 to 602.4)	52.7 (10 to 121.5)	< 0.01	(−)	(−)
SJC, mean (range)	3.7 (1 to 8)	0	< 0.01	(−)	(−)
TJC, mean (range)	6.3 (0 to 17)	0.8 (0 to 3)	< 0.01	(−)	(−)
RF positive (%)	83.3	72.7	0.56	(−)	(−)
ACPA positive (%)	90.0	77.8	0.49	(−)	(−)
DAS28-ESR, mean (range)	4.80 (4.21 to 5.72)	2.33 (0.51 to 3.02)	< 0.01	(−)	(−)
DAS28-CRP, mean (range)	3.84 (3.10 to 4.64)	1.49 (0.98 to 2.2)	< 0.01	(−)	(−)
Medication					
DMARDs (%)	75.0	90.9	0.34	(−)	(−)
Methotrexate (%)	50.0	81.8	0.11	(−)	(−)
Biologics (%)	8.0	18.1	0.51	(−)	(−)
Prednisolone (%)	41.7	0	0.01	(−)	(−)

*ESR* erythrocyte sedimentation rate, *CRP* C-reactive protein, *MMP-3* matrix metalloproteinase-3, *SJC* swollen joint count, *TJC* tender joint count, *RF* rheumatoid factor, *ACPA* anti-cyclic citrullinated peptide antibody, *DAS28-ESR* disease activity score 28 joints using erythrocyte sedimentation rate, *DAS28-CRP* disease activity score 28 joints using C-reactive protein, *DMARDs* disease-modifying anti-rheumatic drugs

This study was approved by the ethics committee of Sagamihara National Hospital and that of St. Marianna University School of Medicine. All of the blood samples were obtained with written informed consent.

### Preparation of serum exosomal proteins

Exosomes were isolated from the serum samples using ExoQuick® (System Bioscience, Mountain View, CA, USA) according to the manufacturer’s instructions. The collected exosomes were dissolved in a cell lysis buffer (30 mM Tris-HCl pH 8.0, 7 M Urea, 2 M Thiourea, 4% 3-(3-cholamidepropyl) dimethylammonio-1-propanesulfonate [CHAPS]). The protein concentrations of the resulting exosome lysates were then determined by the Bradford assay. A portion of the collected exosomes was suspended in phosphate-buffered saline (PBS) for a transmission electron microscopic analysis.

### Transmission electron microscopic analyses

A 5-μl aliquot of PBS-suspended exosomes was placed onto a support grid (Cu, 200 mesh). After 60 s, the grid was washed twice in double distilled water and incubated in 5% aqueous solution of phosphotungstic acid for 10 s. The size and morphology of the collected exosomes were examined using a transmission electron microscope (JEOL Ltd., Tokyo, Japan).

### The 2D-DIGE analysis and protein identification

The exosome lysates were separated by 2D-DIGE, as described previously [25]. In brief, each protein sample was labeled with Cy5 saturation dye. Similarly, 2.5 μg of an internal standard sample (a mixture of equal amounts of all 43 samples) was labeled with Cy3 saturation dye. Then, 2.5 μg of the Cy3-labeled internal standard sample and 2.5 μg of the individual Cy5-labeled protein samples were mixed and subjected to 2D-DIGE. Separated proteins were detected using an image analyzer (Typhoon 9400 Imager; GE Healthcare UK, Buckinghamshire, UK). Acquired gel images were analyzed using the Progenesis software program (PerkinElmer, Waltham, MA, USA), in which the intensity of the protein spots in the gel image of each sample was corrected using that of the internal standard sample.

Proteins were identified by a mass spectrometric analysis as described previously [25]. In brief, the gel fragments that corresponded to protein spots of interest were recovered from 2D gels. Peptides generated by in-gel digestion with trypsin were extracted from the gel fragments. The extracted peptides were then subjected to matrix-assisted laser desorption/ionization time-of-flight/time-of-flight mass spectrometry (Ultraflex; Bruker Daltonics, Bremen, Germany). The determined peptide masses were compiled to allow a search of the National Center for Biotechnology Information protein database using the Mascot software program (Matrix Science, London, UK).

### Detection of TLR3 by Western blotting (WB)

Proteins were extracted from the purified exosomes into lysis buffer (7 M urea, 2 M thiourea, and 4% CHAPS) for WB. The extracted proteins were separated by sodium dodecyl sulfate-polyacrylamide gel electrophoresis (SDS-PAGE) or by 2D electrophoresis (2DE). In 2DE, the protein samples were first subjected to isoelectric focusing (IEF) using gels with the range of p*I*3.0–11.0. The IEF-separated proteins were then further separated by 12.5% SDS-PAGE. The proteins separated by SDS-PAGE or 2DE were transferred onto polyvinylidene difluoride membranes. In WB, the membranes were incubated with rabbit anti-human TLR3 polyclonal antibodies (Abcam, Cambridge, UK) followed by horseradish peroxidase (HRP)-conjugated goat anti-rabbit immunoglobulin G antibodies (Dako, Glostrup, Denmark). The bound antibodies were visualized using ImmunoStar LD (Wako Pure Chemical Industries, Ltd., Tokyo, Japan). In each membrane, the intensity of a blank area in each membrane was defined as 1.0.

### Statistical analyses

The statistical significance of differences in parameters clinical features between the aRA and iRA groups and that of differences in protein spot intensity in 2D-DIGE among the 4 groups was calculated by Student’s *t*-test and a one-way analysis of variance, respectively. The expression of TLR3 fragments (17–18 kDa) in the 4 groups was statistically evaluated by Fisher’s exact test. *P*-values of less than 0.05 were considered to indicate statistical significance.

## Results

### Clinical information of the participants

Clinical information of the 43 participants is shown in Table 1. No significant differences were found between the aRA and iRA groups with regard to age, sex, disease duration, and test items other than markers for inflammatory condition (Table 1). Regarding the medication, the percentage of patients receiving oral corticosteroids in the aRA group (41.7%) was significantly higher than that in the iRA group (0%) (Table 1).

### Isolation of exosomes from serum samples

**Fig. 1:**
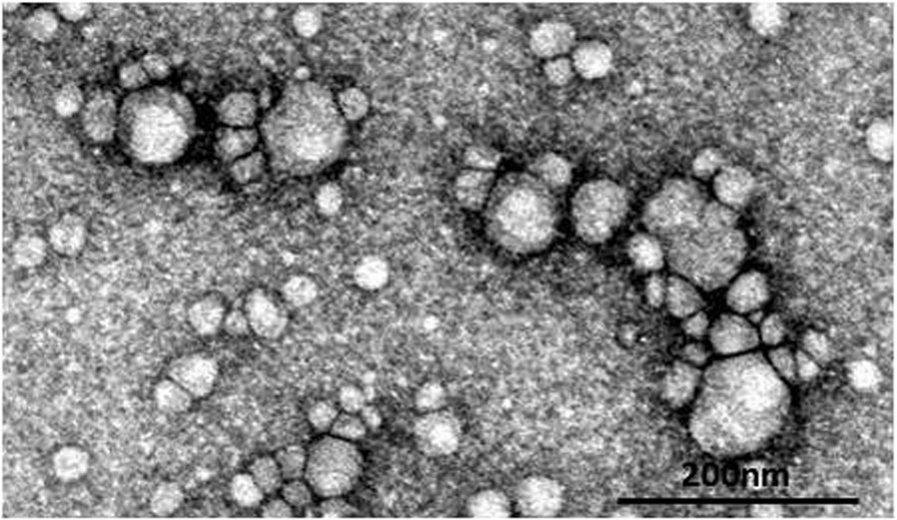
A transmission electron micrograph of exosomes isolated from serum samples. Serum exosomes isolated using Exoquick® were analyzed by transmission electron microscopy. A representative result from the serum exosome samples is shown

We isolated exosomes from serum samples and checked for successful isolation of exosomes using a transmission electron microscopic analysis. Almost all of the isolated extracellular vesicles were found to have a diameter of 50–100 nm, as shown in Fig. 1. The observed sizes were compatible with the definition of exosomes [1, 2].

### The comparison of the protein profiles of serum-derived exosomes among patients with RA and OA and HLs

**Fig. 2:**
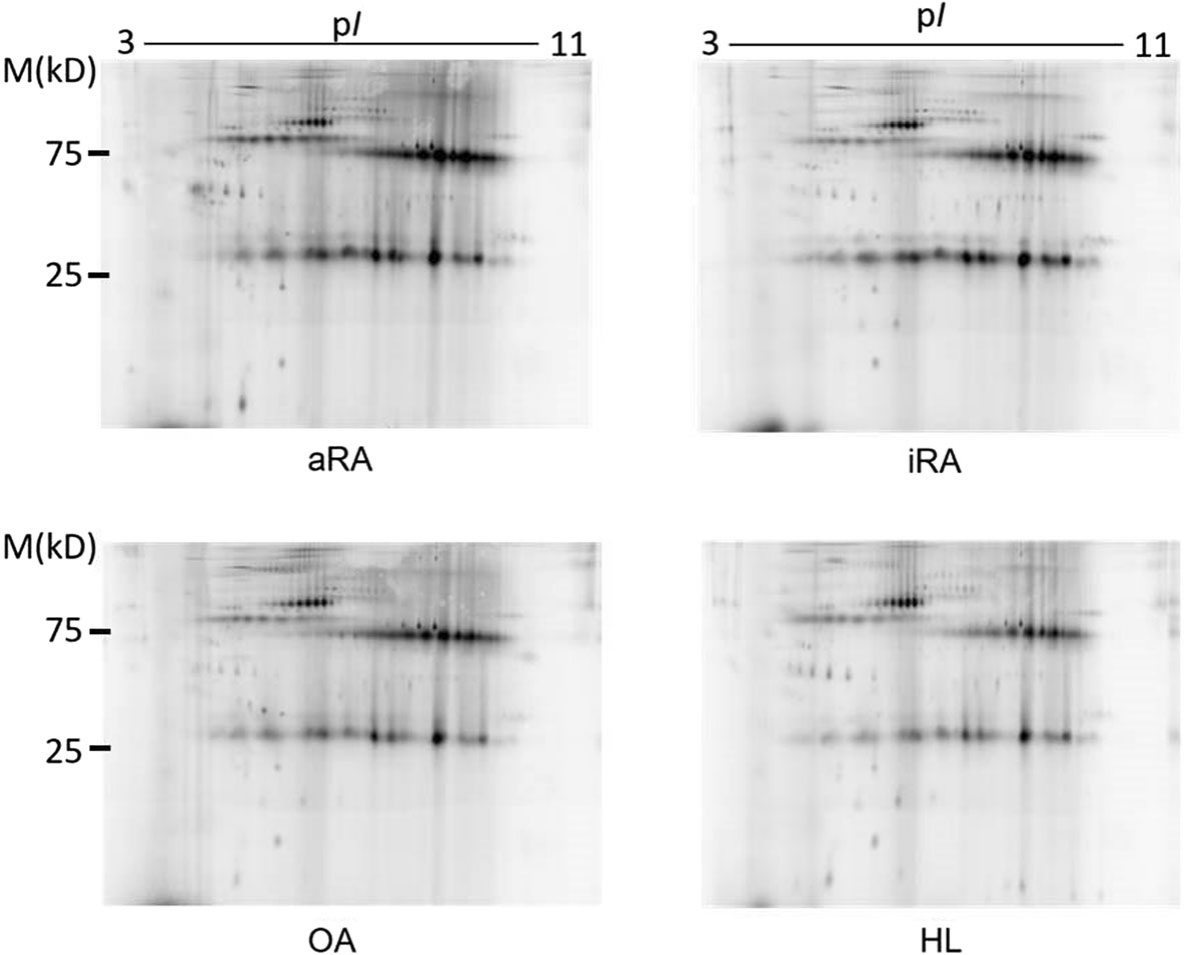
Representative 2D-DIGE images showing protein profiles of exosomes isolated from the serum samples of the aRA, iRA, OA, and HL groups. A representative image from each group is shown

**Fig. 3:**
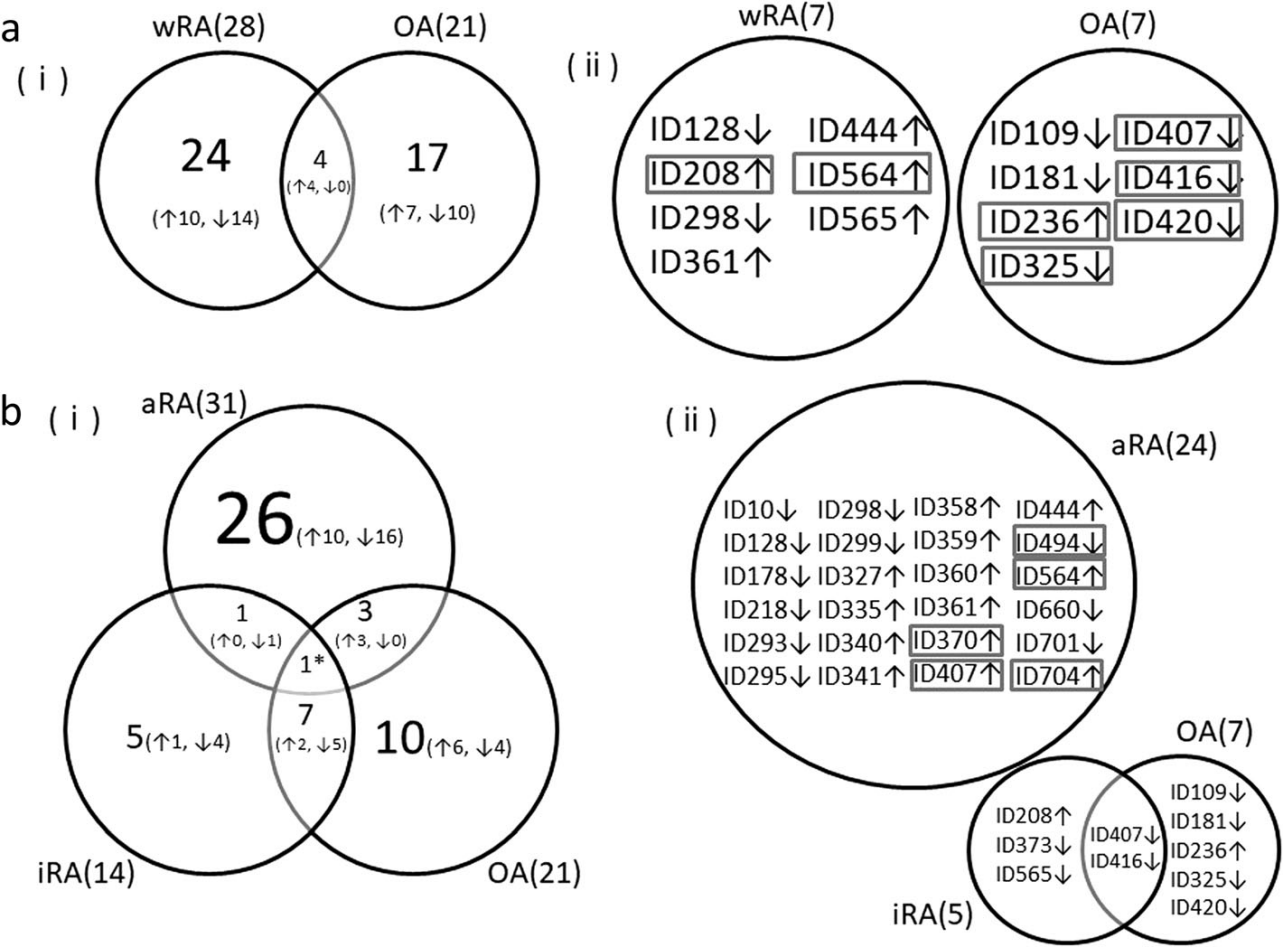
Venn diagrams showing the numbers of protein spots with significantly different intensities between the HL group and each disease group. (**a**) (i) Numbers of protein spots with significantly different intensities between the wRA and HL groups and between the OA and HL groups (*p* < 0.05). Up and down arrows indicate the spots whose intensity was higher and lower, respectively, in the disease groups than that in HL group. (ii) IDs of protein spots with an intensity ≥ ±1.3-fold different in the diagram (i). IDs of protein spots with a significantly different intensity between the wRA and OA groups are boxed. Up and down arrows indicate the spots whose intensity was higher and lower, respectively, compared to the intensity of the identical spots in the HL group. (**b**) (i) Numbers of protein spots with significantly different intensities between the aRA and HL groups, between the iRA and HL groups, and between the OA and HL groups (*p* < 0.05). Up and down arrows indicate the spots whose intensity was higher and lower, respectively, in the disease groups than that in HL group. The spots with “*” showed a higher intensity in the aRA group and a lower intensity in the iRA and OA groups than in the HL group. (ii) IDs of protein spots with an intensity ≥ ±1.3-fold different in the diagram (i). IDs of protein spots with a significantly different intensity between the aRA and OA groups and between the aRA and iRA groups are boxed. Up and down arrows indicate the spots whose intensity was higher and lower, respectively, compared to the intensity of the identical spots in the HL group

We investigated the protein profiles of exosomes in each of the 43 serum samples by 2D-DIGE. As a result, a total of 204 protein spots were detected on the gels, as shown in Fig. 2. The spot intensities in each group are shown in Additional file 1: Table S1. We first compared the intensity of the separated protein spots between the whole RA (wRA; aRA + iRA) and HL groups and between the OA and HL groups. In the comparison between the wRA and HL groups, 28 of the 204 protein spots showed significantly different intensities (*p* < 0.05) (Table 2A). Of these 28 spots, 7 showed a difference in intensity exceeding ±1.3-fold (Table 2A). In the comparison between the OA and HL groups, 21 protein spots showed different intensities (*p* < 0.05) (Table 2D). Of these 21 spots, 7 showed a difference in intensity exceeding ±1.3-fold (Table 2D). As shown in Fig. 3a-(i), only 4 spots overlapped between the 28 spots that differed in the wRA group and the 21 spots that differed in the OA group (14% and 19%, respectively). Furthermore, no spots overlapped between these 2 groups when we focused on the spots with a difference in intensity exceeding ±1.3-fold (Fig. 3a-[ii]). These results indicate that the protein profiles of exosomes are quite different between the wRA and OA groups.

**Table 2:** Differences in the exosomal protein profiles among the disease categories detected by 2D-DIGE

A. wRA vs. HL			
Total number of detected protein spots	Number of spots with different intensities (*p* < 0.05)	fold difference (wRA/HL)	number of spots
204	28	X ≥ 1.3	5
		1.3 > X > −1.3	21
		−1.3 ≥ X	2
B. aRA vs. HL			
Total number of detected protein spots	Number of spots with different intensities (*p* < 0.05)	fold difference (aRA/HL)	number of spots
204	31	X ≥ 1.3	13
		1.3 > X > −1.3	7
		− 1.3 ≥ X	11
C. iRA vs. HL			
Total number of detected protein spots	Number of spots with different intensities (*p* < 0.05)	fold difference (iRA/HL)	number of spots
204	14	X ≥ 1.3	1
		1.3 > X > −1.3	9
		−1.3 ≥ X	4
D. OA vs. HL			
Total number of detected protein spots	Number of spots with different intensities (*p* < 0.05)	fold difference (OA/HL)	number of spots
204	21	X ≥ 1.3	1
		1.3 > X > −1.3	14
		−1.3 ≥ X	6

*wRA* whole RA, *aRA* active RA, *iRA* inactive RA, *OA* osteoarthritis, *HL* healthy donor

We next compared the protein profiles between the aRA and HL groups and between the iRA and HL groups. On comparing the aRA and HL groups, 31 spots showed different intensities (*p* < 0.05) (Table 2B). Of these 31 spots, 24 showed a difference in intensity exceeding ±1.3-fold (Table 2B). On comparing the iRA and HL groups, 14 protein spots showed different intensities (*p* < 0.05) (Table 2C). Of these 14 spots, 5 showed a difference in intensity exceeding ±1.3-fold (Table 2C). Of note, as shown in Fig. 3b-(i), only 2 spots overlapped between the 31 spots that differed in the aRA group and the 14 spots that differed in the iRA group (6.5% and 14%, respectively). Furthermore, no spots overlapped between the 2 groups when we focused on the spots with a difference in intensity exceeding ±1.3-fold (Fig. 3b-[ii]). These results indicate that the protein profiles of exosomes differ markedly between the aRA and iRA groups. In addition, only 4 spots overlapped between the 31 spots that differed in the aRA group and the 21 spots that differed in the OA group (13% and 19%, respectively) (Fig. 3b-[i]). No spots overlapped between the 2 groups when we focused on the spots with a difference in intensity exceeding ±1.3-fold (Fig. 3b-[ii]). These results indicate that the protein profiles of exosomes differ markedly between the aRA and OA groups. In contrast, 6 spots overlapped between the 14 spots that differed in the iRA group and the 21 spots that differed in the OA group (43% and 29%, respectively) (Fig. 3b-[i]). The location of spots that showed an intensity with more than ±1.3-fold difference (*p* < 0.05) compared to that in the HL group were marked on the 2D-DIGE images of the aRA, iRA, and OA groups (Additional file 1: Figure S1).

### Identification of proteins in the spots with different intensity

We then compared the spot intensity of the 14 spots shown in Fig. 3a-(ii) between the wRA and OA groups. As a result, 7 spots (ID208, ID564, ID407, ID416, ID420, ID236, and ID325, rectangle in Fig. 3a-[ii]) showed different intensities (*p* < 0.05) between the wRA and OA groups. Similarly, we compared the spot intensity of the 34 spots (including 2 overlapping spots) shown in Fig. 3b-(ii) between the aRA and OA groups and between the aRA and iRA groups. As a result, 5 spots (ID370, ID407, ID494, ID564, and ID704, rectangle in Fig. 3b[ii]) showed different intensity (*p* < 0.05) between the aRA and OA groups and between the aRA and iRA groups.

**Fig. 4:**
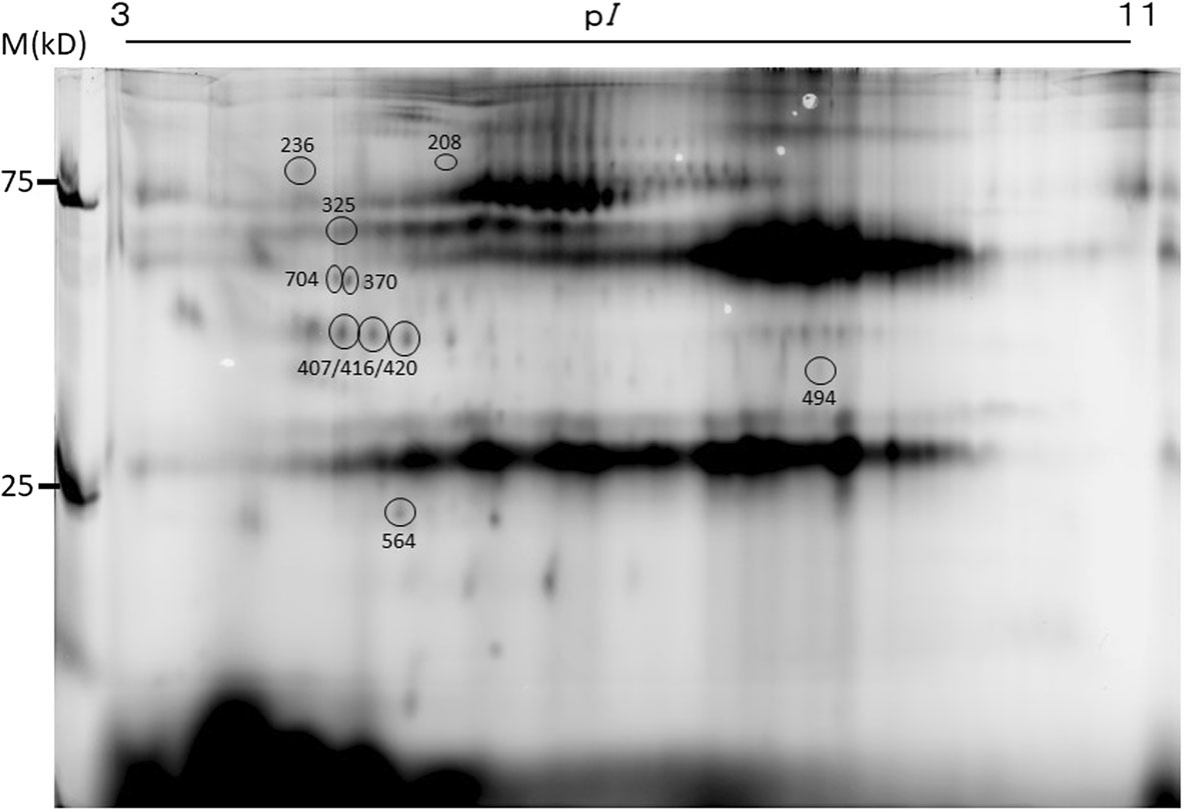
Location and IDs of exosomal protein spots subjected to identification

We tried to identify the protein names of the former 7 spots and the latter 5 spots (actually 10 spots because of 2 overlapped spots [ID407 and ID564]) by a mass spectrometric analysis (Fig. 4). As a result, we were able to name 6 out of the 10 protein spots, as listed in Table 3. ID208 (increased only in the wRA group) was found to be a membrane-bound isoform of Pro-neuregulin-3. ID564 (increased in the wRA and aRA groups) was found to be TLR3. ID370 (increased only in the aRA group) was found to be alpha-1-antitrypsin. ID494 (decreased only in the aRA group) was found to be keratin of type II cytoskeletal 1. ID236 (increased only in the OA group) was found to be cathepsin F. ID 325 (decreased only in the OA group) was found to be Ig alpha-2 chain C region. We confirmed that these 6 identified proteins were registered in Vesiclepedia, a database of extracellular vesicle components [26].

**Table 3:** Proteins identified by mass spectrometry

Spot ID	Observed		Fold difference				Protein	Theoretical		Masscot Score	Coverage (%)	Confirmed Sequences (Masscot ion score)	Search log
	MW	p*I*	wRA		OA	HL		MW	p*I*				
			aRA	iRA									
208	90	5.3	1.4*		1.0	1.0	Pro-neuregulin-3, membrane-bound isoform	78	7.8	61	12	^388^QAKQIQEQLK^397^(19)	38,220
							NRG3_HUMAN						
236	87	5.1	1.1		1.3*	1.0	Cathepsin F	53	8.5	26	3	^103^TLLCSFQVLDELGRHVLLR^121^(26)	38,210
							CATF_HUMAN						peptide summary
325	65	5.1	0.9		0.7*	1.0	Ig alpha-2 chain C region	37	5.7	132	10	^251^WLQGSQELPR^260^(53)	38,207
							IGHA2_HUMAN					^270^QEPSQGTTTFAVTSILR^286^(70)	
370	53	5.1	1.3*	0.9	0.9	1.0	Alpha-1-antitrypsin	47	5.4	170	33	^50^ITPNLAEFAFSLYR^63^(79)	38,190
							A1AT_HUMAN					^216^GKWERPFEVK^225^(10)	
494	40	7.0	0.7*	0.9	0.9	1.0	Keratin, type II cytoskeletal 1	66	8.2	62	9	^212^WELLQQVDTSTR^223^(14)	38,206
							K2C1_HUMAN					^224^THNLEPYFESFINNLR^239^(41)	
564	24	5.2	6.3*	1.2	1.1	1.0	Toll-like receptor 3	104	6.7	68	19	^532^LEILDLQHNNLAR^544^(7)	38,215
							TLR3_HUMAN						

*MW* molecular weight, *wRA* whole RA, *aRA* active RA, *iRA* inactive RA, *OA* osteoarthritis, *HL* healthy donor

Fold differences are shown with the intensity of the protein spots in HLs defined as 1.0. Asterisks indicate that the spot intensity is significantly different (*p* < 0.05) from that in HLs

**Fig. 5:**
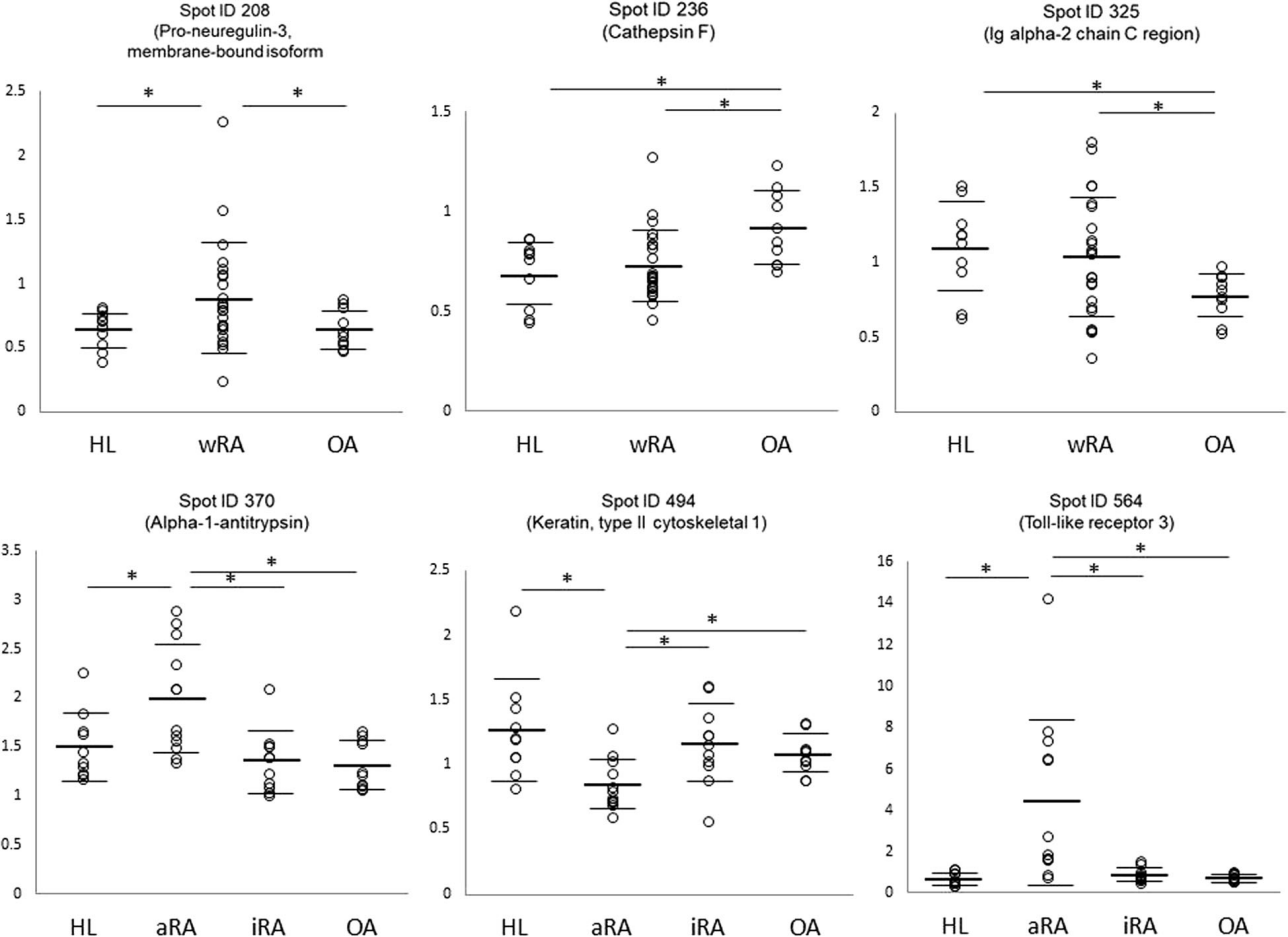
Differences in the relative spot intensities of the identified proteins. Open circles show the relative intensity of individual samples. Bars show the mean ± SD. *, *p* < 0.05

Among these six protein spots, the difference in ID564 (assigned as TLR3) was most drastic, as shown in Fig. 5. Since the observed molecular weight (MW) (24 kD) of ID564 was rather small compared to the theoretical MW (104 kD) of full-length TLR3, ID564 was thought to be a part of TLR3. Mass spectrometry detected a peptide corresponding to the 532–544 amino acid residues of TLR3 (Table 3), indicating that the spot ID564 was a middle part of TLR3.

### Detection of the TLR3 fragment by 1DE- and 2DE-WB

**Fig. 6:**
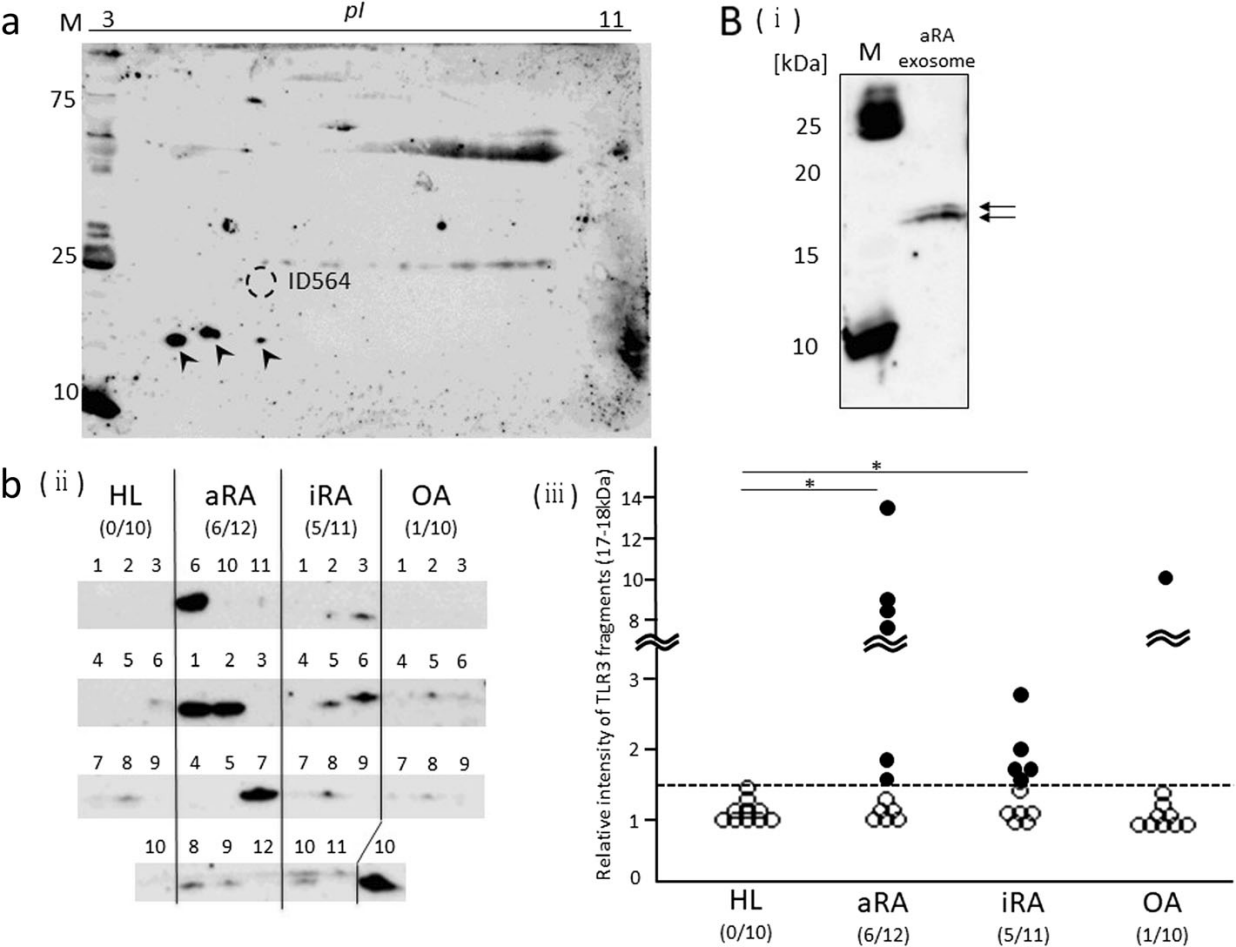
Detection of TLR3 in the serum-derived exosome protein. (**a**) TLR3 was detected by 2DE-WB from the exosomal protein sample mixture from 4 aRA patients (aRA 1, 6, 10, and 11) with a relatively high ID564 intensity. Arrowheads, TLR3 fragments (17–18 kDa). (**b**) (i) The TLR3 fragments (17–18 kDa) in the exosomal protein samples described in (**a**) were detected by 1DE-WB. (ii) The TLR3 fragments (17–18 kDa) in the exosomal protein samples from the HLs (*n* = 10) and patients with aRA (*n* = 12), iRA (*n* = 11), and OA (*n* = 10) were detected by 1DE-WB. (iii) The intensity of the bands was measured using a densitometer. The intensity of the blank area in each membrane was defined as 1.0. The obtained relative expression of the TLR3 fragments (17–18 kDa) was compared among the 4 groups. The dotted line indicates the mean + 2.5SD of the band intensity in the HL group. The intensity of the TLR3 fragments (17–18 kDa) was higher in 6 of the 12 aRA samples, 5 of the 11 iRA samples, and 1 of the 10 OA samples than the mean of that in HL groups + 2.5SD (filled circles). The ratios of samples with a high intensity of TLR3 fragments (17–18 kDa) were significantly higher in the aRA and iRA groups than in the HL group. * *p* < 0.05

We tried to detect the TLR3 fragment (24 kDa) corresponding to the ID564 spot using 2DE-WB and a mixture of 4 aRA exosomal protein samples with a relatively high intensity of ID564 using antibodies raised against the human TLR3 fragment of amino acid 464–647. However, the ID564 spot was not detected by the antibody; instead, 3 protein spots of 17–18 kDa were detected (Fig. 6a). We therefore focused on the TLR3 fragments (17–18 kDa) and compared the expression of these fragments among the HL (*n* = 10), aRA (*n* = 12), iRA (*n* = 11), and OA (*n* = 10) groups by 1DE-WB. The 17- to 18-kDa TLR3 spots were ultimately detected as doublet bands on 1DE-WB (Fig. 6b [i]). In 6 of the 12 aRA samples, 5 of the 11 iRA samples and 1 of the 10 OA samples, the band intensity of the TLR3 fragments was higher than the mean of that in the HL group + 2.5 standard deviations (Fig. 6b [ii] and [iii]). In the aRA and iRA groups, the ratios of samples with a high band intensity of TLR3 fragments (17–18 kDa) were significantly higher than in the HL group (Fig. 6b [ii] and [iii]). These results suggest that patients with RA more frequently have large amounts of TLR3 fragments (17–18 kDa) in serum-derived exosomes than HLs.

## Discussion

To our knowledge, this is the first report to compare the protein profiles of serum exosomes among patients with RA, those with OA, and HLs.

In the aRA group, ID564, identified as TLR3, showed about 6-fold higher intensity than the other groups. Given the observed small MW (24 kDa) of ID564 and the mass spectrometric data, ID564 was thought to represent a middle part fragment of TLR3. Furthermore, patients with RA more frequently had large amounts of the TLR3 fragments (17–18 kDa) in serum-derived exosomes than HLs.

TLR3 is a member of the TLR family of pattern recognition receptors of the innate immune system [27]. TLR3 is an endosomal TLR that recognizes double-stranded RNA (dsRNA), a molecular pattern associated with viral infection [27, 28]. TLR3 signal activates transcription factors of NF-κB and interferon regulatory factor (IRF) 3, which leads to the secretion of type I interferons and proinflammatory cytokines, such as IL-6 and IL-8 [27, 28]. In the intracellular trafficking and maturation of TLR3, a precursor form of TLR3 is transported to endosomes through the endoplasmic reticulum (ER) and Golgi apparatus [29]. In the endosomes, the precursor form of TLR3 is cleaved to produce a C-terminal 70-kDa fragment of TLR3 that can deliver signals [29]. The amino acid sequences of ID564 identified by the mass spectrometric analysis were located in the range of the C-terminal 70-kDa fragment (Table 3). We therefore considered that the 24-kDa fragments of TLR3 had been generated by additional proteolytic cleavage of the C-terminal 70-kDa fragment in endosomes and then taken up into exosomes after the cleavage.

Several reports have suggested that the expression of TLR3 and the activation of its pathways are associated with the pathogenesis of RA. For example, TLR3 was found to be more highly expressed in RA synovial tissue than in OA synovial tissue [30]. The stimulation of cultured fibroblast-like synoviocytes (FLSs) with poly (I-C), a synthetic TLR3 ligand, up-regulated the TLR3 expression in FLSs [30]. RNA released from necrotic synovial fluid cells of RA patients activated the TLR3 pathway in FLSs and up-regulated the expression of IFN-β mRNA and the production of IL-6 [30]. It was recently reported that poly (I:C) of a TLR3 ligand associated with extracellular vesicles (EVs) was able to selectively activate antiviral and proinflammatory responses in synovial fibroblasts [31]. In addition, it has been reported that the expression of TLR3 is increased in the spleen of rats with collagen-induced arthritis as well as in those with pritane-induced arthritis, and that the increased expression of TLR3 was decreased by treatment with MTX [32]. Thus, TLR3, which is highly expressed in synoviocytes and possibly in the spleen of patients with active RA as well, is thought to play a role in the production of proinflammatory cytokines. The markedly high levels of the TLR3 fragment in serum exosomes may be caused by the high expression of TLR3 in FLSs in patients with active RA. From this viewpoint, the 24- and the 17–18 kDa TLR3 fragments in serum exosomes may be useful markers of the activation of FLSs in patients with RA.

While the TLR3 fragments (17–18 kDa and 24 kDa) are likely non-functional, they may still have some pathological significance. We previously reported that a degradation product of Apo-AI has a biological function of promoting IL-8 secretion from cells [33]. The TLR3 fragments detected in the present study might therefore perform some biological functions that differ from those of full-length TLR3. Further studies will be needed to investigate this possibility.

A membrane-bound isoform of pro-neuregulin-3 showed 1.4-fold higher expression in the wRA group than in the OA and HL groups (Table 3, Fig. 5). Pro-neuregulin-3 is a precursor of neureglin-3, which is a ligand for ErbB4, a member of the epidermal growth factor receptor (EGFR) family [34]. It has been reported that the binding of neureglin-3 to ErbB4 promotes the proliferation, migration, and differentiation of neuroblasts [34, 35]. Although little is known about the involvement of neuregulin in the pathogenesis of rheumatic diseases, it has been reported that EGFR is expressed in fibroblasts and vascular endothelial cells and that EGFR signaling induces FLS proliferation and cytokine production in patients with RA [36]. Given the high expression of pro-neuregulin-3 in serum-derived exosomes of patients with RA detected in our study, the neuregulin-3-ErbB4 pathway may be activated to promote proliferation in FLSs. Furthermore, pro-neuregulin-3 in serum exosomes may be converted to neuregulin-3 and act as a ligand for ErbB4 in FLSs. Further studies are needed to elucidate these points.

The intensity of the spot ID236, identified as cathepsin F, was higher in the OA group than in the other three groups. Cathepsin F is a member of the papain family cysteine proteinases [37]. Given that another member of cathepsin K was reported to be involved in the pathogenesis of OA [38, 39], cathepsin F might also be involved in the pathogenesis of OA. This point needs to be investigated in the future.

The observed MWs of the proteins identified in this study were slightly different from their theoretical MWs (Table 3). Post-translational modifications, such as glycosylation, have been reported for the spots ID208 (pro-neuregulin-3, membrane-bound isoform), ID236 (cathepsin F), and ID325 (Ig alpha-2 chain C region), which may have contributed to this discrepancy in MW findings [40–42]. For spot ID494 (Keratin, type II cytoskeletal), the observed MW was smaller than the theoretical MW, suggesting that ID494 might be a keratin degradate, similar to the case of TLR3.

Exosomal lymphatic vessel endothelial hyaluronic acid receptor-1 (LYVE-1) was very recently reported to be a candidate marker of RA activity by a 2D-LC-MS/MS (two-dimensional liquid chromatography-tandem mass spectrometry) analysis of serum exosomal proteins from RA patients with complete remission and those with non-complete remission [19]. However, in that study, none of the six proteins we identified in our own were found to be candidate markers of RA activity [19]. Similarly, LYVE-1 was not identified as a differently expressed protein in our study [19]. This difference may be due to differences in the serum donors (we analyzed exosomal samples from OA patients and HLs in addition to patients with RA) and different methods used in these two studies (2D-LC-MS/MS vs. 2D-DIGE followed by MS/MS).

In addition, we isolated serum exosomes using Exoquick®, which was useful to isolate exosomes from a large number of blood samples. However, similarly as other methods, contamination of serum proteins cannot be completely avoided. It would be needed to investigate characters and functions of the individual identified proteins as exosomal proteins.

## Conclusions

In the present study, we demonstrated that the serum exosomes of patients with aRA have different protein profiles from those of patients with iRA, patients with OA, and HLs. The 24- and the 17- to 18-kDa TLR3 fragments, which were found to be markedly increased in patients with aRA, may reflect the inflammatory condition of FLSs. Our findings may aid in the clarification of the roles of exosomes in the pathophysiology of RA.

## Additional file

Additional file 1: **Figure S1.** Location of differently enriched protein spots. **Table S1.** Averages of spot intensities of each group. (DOCX 101 kb)

## Abbreviations

2D-DIGE: Two-dimensional differential image gel electrophoresis
2D-LC-MS/MS: Two-dimensional liquid chromatography-tandem mass spectrometry
ACPA: Anti-cyclic citrullinated peptide antibody
APC: Antigen-presenting cell
aRA: Active RA
CHAPS: 3-(3-cholamidepropyl) dimethylammonio-1-propanesulfonate
CRP: C-reactive protein
DAS28-CRP: Disease activity score 28 joints using C-reactive protein
DAS28-ESR: Disease activity score 28 joints using erythrocyte sedimentation rate
DC: Dendritic cell
DMARDs: Disease-modifying anti-rheumatic drugs
dsRNA: Double-stranded RNA
EGFR: Epidermal growth factor receptor
ER: Endoplasmic reticulum
ESR: Erythrocyte sedimentation rate
FLS: Fibroblast-like synoviocyte
HL: Healthy control
ICAM: Intercellular adhesion molecule
IFN: Interferon
IL: Interleukin
iRA: Inactive RA
IRF: Interferon regulatory factor
LYVE-1: Lymphatic vessel endothelial hyaluronic acid receptor-1
MHC: Major histocompatibility complex
MMP: Matrix metalloproteinase
MS: Mass spectrometry
MTX: Methotrexate
MVBs: Multivesicular bodies
MW: Molecular weight
NF-κB: Nuclear factor-kappa B
OA: Osteoarthritis
PBS: Phosphate-buffered saline
PD-L1: Programmed death-ligand 1
RA: Rheumatoid arthritis
RF: Rheumatoid factor
SASP: Salazosulfapyridine
SJC: Swollen joint count
TJC: Tender joint count
TLR: Toll-like receptor
TNF: Tumor necrosis factor
wRA: Whole RA
