# Supplementary material for: A proteomic analysis of serum-derived exosomes in rheumatoid arthritis
Source: BMC Rheumatol. 2018 Nov 27;2:35. doi: 10.1186/s41927-018-0041-8 (PMC6390805; doi:10.1186/s41927-018-0041-8)
Supplement: Supplementary file 1 — Figure S1. Location of differently enriched protein spots. Table S1. Averages of spot intensities of each group. (DOCX 101 kb) [file 41927_2018_41_MOESM1_ESM.docx]

**Additional file 1**

**Figure S1. Location of differently enriched protein spots.**

Spots that showed an intensity ≥ ±1.3-fold different with statistical significance from that in the HL group were marked.

**
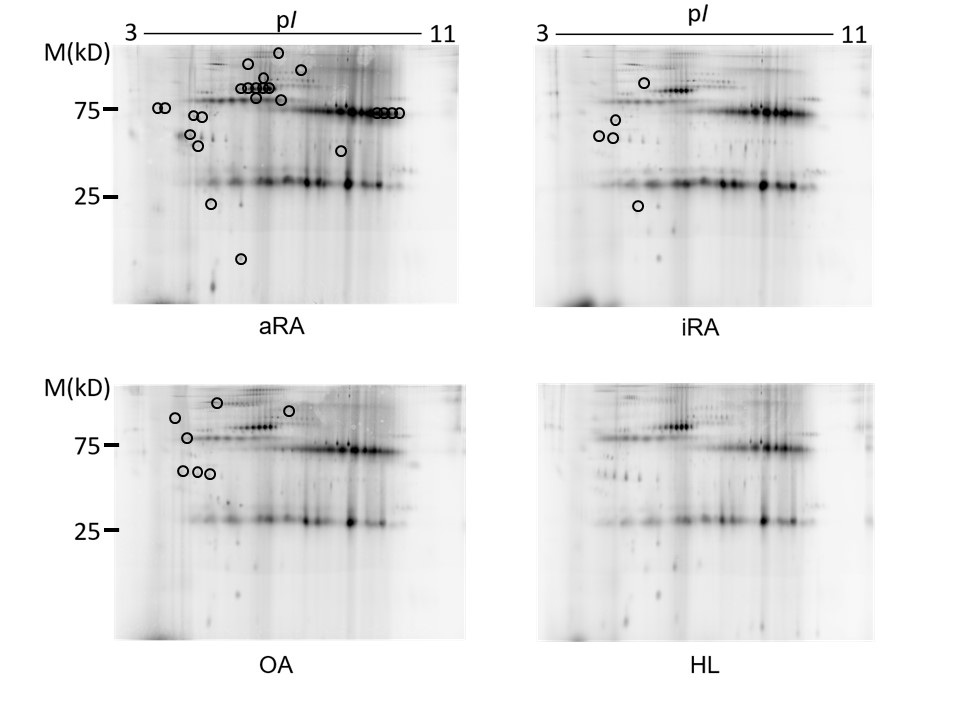
**

**Table S1. Averages of spot intensities of each group**

| Spot ID | aRA | iRA | OA | HL |
| --- | --- | --- | --- | --- |
| 10 | 0.48271 | 0.59868 | 0.634491 | 0.667201 |
| 11 | 0.48744 | 0.61868 | 0.579156 | 0.582466 |
| 39 | 0.45034 | 0.52305 | 0.535925 | 0.421706 |
| 46 | 0.55148 | 0.45794 | 0.60975 | 0.546479 |
| 103 | 1.58153 | 1.31043 | 1.174004 | 1.416674 |
| 105 | 1.74892 | 1.3591 | 1.330268 | 1.628913 |
| 106 | 1.78046 | 1.38432 | 1.383888 | 1.641395 |
| 108 | 1.72195 | 1.33025 | 1.253664 | 1.580232 |
| 109 | 1.53708 | 1.29822 | 1.216103 | 1.60599 |
| 111 | 1.40132 | 1.21422 | 1.224499 | 1.498921 |
| 112 | 1.23342 | 1.14726 | 1.250585 | 1.35316 |
| 115 | 0.88314 | 1.01466 | 1.101695 | 1.018402 |
| 116 | 0.94633 | 1.0496 | 1.203735 | 1.092822 |
| 117 | 0.79982 | 0.92581 | 1.049708 | 0.884617 |
| 118 | 0.68817 | 0.81097 | 0.948901 | 0.838069 |
| 125 | 0.54538 | 0.60558 | 0.656365 | 0.643287 |
| 128 | 0.51618 | 0.56638 | 0.679727 | 0.733431 |
| 142 | 0.93033 | 1.24521 | 0.9327 | 1.039383 |
| 158 | 1.557 | 1.41213 | 1.42674 | 1.579804 |
| 160 | 1.559 | 1.33608 | 1.264536 | 1.556854 |
| 161 | 1.36227 | 1.38859 | 1.274654 | 1.574371 |
| 163 | 1.56777 | 1.4768 | 1.330828 | 1.595814 |
| 177 | 0.17162 | 0.17406 | 0.222105 | 0.217322 |
| 178 | 0.54772 | 0.66287 | 0.662926 | 0.730739 |
| 181 | 0.7909 | 0.67885 | 0.616798 | 0.840281 |
| 183 | 0.85232 | 0.69899 | 0.653274 | 0.83371 |
| 186 | 0.43893 | 0.32317 | 0.360403 | 0.413715 |
| 208 | 0.81476 | 0.97744 | 0.649843 | 0.645639 |
| 218 | 0.29935 | 0.34563 | 0.352453 | 0.403441 |
| 219 | 0.33316 | 0.4027 | 0.409029 | 0.408421 |
| 220 | 0.40488 | 0.52852 | 0.469068 | 0.450359 |
| 222 | 0.93467 | 1.11392 | 0.814599 | 0.99815 |
| 236 | 0.70687 | 0.76818 | 0.921595 | 0.697704 |
| 237 | 0.6635 | 0.57835 | 0.634014 | 0.665421 |
| 238 | 0.5803 | 0.51806 | 0.626623 | 0.615193 |
| 239 | 0.63582 | 0.52943 | 0.630462 | 0.661769 |
| 241 | 0.63717 | 0.52349 | 0.633225 | 0.609834 |
| 242 | 0.52015 | 0.41743 | 0.482587 | 0.515743 |
| 243 | 0.60447 | 0.47032 | 0.570145 | 0.515681 |
| 257 | 1.05982 | 1.15053 | 0.941229 | 0.796416 |
| 259 | 1.29275 | 1.39754 | 1.20017 | 0.995399 |
| 261 | 1.29795 | 1.36576 | 1.124941 | 0.989659 |
| 263 | 0.97846 | 1.15211 | 0.83503 | 0.609512 |
| 265 | 1.13882 | 1.5326 | 1.110773 | 0.947903 |
| 266 | 1.12412 | 1.34083 | 1.053701 | 0.904459 |
| 267 | 0.9725 | 1.33937 | 0.913813 | 0.842054 |
| 268 | 1.02893 | 1.1918 | 0.968975 | 0.795278 |
| 285 | 0.48349 | 0.63533 | 0.471895 | 0.637005 |
| 286 | 0.55582 | 0.64773 | 0.527761 | 0.614492 |
| 287 | 0.37873 | 0.52839 | 0.451069 | 0.508766 |
| 288 | 0.47842 | 0.55268 | 0.474558 | 0.588023 |
| 290 | 0.43376 | 0.51251 | 0.465154 | 0.554304 |
| 293 | 0.42489 | 0.46827 | 0.463996 | 0.564685 |
| 294 | 0.36425 | 0.42327 | 0.389561 | 0.356671 |
| 295 | 0.40798 | 0.47187 | 0.479932 | 0.560276 |
| 298 | 0.40764 | 0.4662 | 0.502785 | 0.584639 |
| 299 | 0.41794 | 0.48466 | 0.501318 | 0.544598 |
| 300 | 0.26943 | 0.35134 | 0.332932 | 0.309768 |
| 305 | 1.2029 | 1.02485 | 0.885272 | 1.140119 |
| 310 | 1.27301 | 1.13278 | 1.019915 | 1.278396 |
| 311 | 1.0619 | 1.14116 | 1.106353 | 1.317625 |
| 312 | 1.19798 | 1.08702 | 1.113264 | 1.282293 |
| 313 | 1.12192 | 1.10873 | 1.039541 | 1.299413 |
| 315 | 1.14627 | 1.41268 | 1.167036 | 1.013441 |
| 316 | 1.24953 | 1.2402 | 1.317867 | 1.134836 |
| 317 | 1.35698 | 1.39566 | 1.45062 | 1.354802 |
| 318 | 1.38879 | 1.52572 | 1.71124 | 1.599352 |
| 319 | 1.53489 | 1.5741 | 1.842869 | 1.66639 |
| 320 | 1.39295 | 1.67841 | 1.986883 | 1.848874 |
| 324 | 1.05069 | 0.96591 | 0.922779 | 0.818658 |
| 325 | 0.89207 | 1.19265 | 0.783965 | 1.101381 |
| 326 | 1.10349 | 0.97854 | 0.804213 | 0.917211 |
| 327 | 1.33889 | 1.11746 | 0.909044 | 1.020924 |
| 328 | 1.42103 | 1.09417 | 1.022746 | 1.199913 |
| 329 | 1.08739 | 1.04877 | 0.886234 | 1.024496 |
| 330 | 1.4324 | 1.02344 | 0.870454 | 1.036813 |
| 331 | 0.97124 | 1.09003 | 0.902094 | 1.1631 |
| 332 | 1.03351 | 1.09902 | 0.873862 | 1.059178 |
| 333 | 1.2266 | 0.95739 | 0.877905 | 0.905457 |
| 334 | 1.06263 | 1.16785 | 0.887049 | 1.149721 |
| 335 | 1.11603 | 0.87064 | 0.814091 | 0.805477 |
| 336 | 1.12102 | 1.09611 | 1.261978 | 1.076945 |
| 337 | 1.30579 | 1.15765 | 1.468241 | 1.237899 |
| 338 | 0.9398 | 0.9926 | 1.019113 | 0.89181 |
| 339 | 0.77765 | 0.66213 | 0.609453 | 0.709359 |
| 340 | 1.25731 | 1.04324 | 1.145088 | 0.955981 |
| 341 | 1.28884 | 1.03629 | 1.239723 | 0.956838 |
| 343 | 0.96215 | 0.97432 | 1.027153 | 1.182521 |
| 344 | 0.92058 | 0.75715 | 0.866455 | 0.877241 |
| 345 | 0.74481 | 0.8975 | 0.918755 | 0.840342 |
| 346 | 0.8728 | 0.95025 | 0.970839 | 1.065056 |
| 348 | 0.85868 | 0.94027 | 0.988013 | 1.047329 |
| 349 | 0.78671 | 0.8851 | 0.90608 | 0.956974 |
| 350 | 0.83452 | 0.90985 | 0.914796 | 0.990347 |
| 351 | 1.01607 | 0.98198 | 0.959075 | 1.376817 |
| 352 | 0.84143 | 0.9187 | 0.893953 | 0.933468 |
| 353 | 0.85026 | 0.93101 | 0.901505 | 0.937421 |
| 354 | 0.85353 | 0.89647 | 0.885924 | 0.87196 |
| 355 | 0.88336 | 0.85155 | 0.879516 | 0.850297 |
| 356 | 0.95246 | 0.90383 | 0.917768 | 0.825388 |
| 357 | 1.07867 | 0.97018 | 0.991063 | 0.910474 |
| 358 | 1.20533 | 1.03914 | 1.066664 | 0.921333 |
| 359 | 1.36503 | 1.15036 | 1.159785 | 1.013985 |
| 360 | 1.3172 | 1.08282 | 1.110626 | 0.946806 |
| 361 | 1.44416 | 1.16441 | 1.178064 | 0.994601 |
| 366 | 1.16274 | 0.83979 | 0.912835 | 0.973687 |
| 368 | 1.38986 | 1.49058 | 1.213471 | 1.312546 |
| 370 | 1.9918 | 1.3551 | 1.317115 | 1.509687 |
| 371 | 1.86163 | 1.37102 | 1.333478 | 1.589643 |
| 372 | 1.0129 | 1.29884 | 1.385042 | 1.610984 |
| 373 | 1.95218 | 1.27168 | 1.419613 | 1.761001 |
| 375 | 0.75561 | 0.75919 | 0.842721 | 0.811405 |
| 376 | 2.03575 | 1.57955 | 1.201775 | 1.541444 |
| 378 | 1.24021 | 1.57099 | 1.3917 | 1.209089 |
| 379 | 1.15162 | 1.27006 | 1.371401 | 1.308567 |
| 380 | 1.2615 | 1.47277 | 1.598172 | 1.423034 |
| 381 | 0.93616 | 0.84861 | 0.968008 | 0.94459 |
| 385 | 0.89567 | 0.91285 | 1.018729 | 0.915857 |
| 389 | 0.39697 | 0.37639 | 0.347048 | 0.290558 |
| 390 | 0.70742 | 0.80653 | 0.70176 | 0.771352 |
| 399 | 1.45021 | 0.91598 | 0.824436 | 1.13308 |
| 402 | 2.0676 | 0.91064 | 1.345655 | 1.035519 |
| 407 | 2.58477 | 1.054 | 1.034634 | 1.691754 |
| 416 | 2.45302 | 1.03147 | 0.958851 | 1.765094 |
| 420 | 2.11655 | 1.06636 | 0.883983 | 1.805506 |
| 422 | 1.02036 | 1.22441 | 1.220285 | 1.200524 |
| 423 | 1.35102 | 1.61074 | 1.527003 | 1.526646 |
| 425 | 1.08828 | 1.31029 | 1.447043 | 1.33951 |
| 426 | 1.20338 | 1.48089 | 1.564372 | 1.43697 |
| 427 | 1.50877 | 1.02548 | 0.807506 | 1.595068 |
| 429 | 1.5896 | 1.59898 | 1.444837 | 1.450408 |
| 430 | 1.5067 | 1.54523 | 1.475657 | 1.437746 |
| 431 | 1.3561 | 1.32581 | 0.945955 | 1.648603 |
| 433 | 1.60835 | 1.64489 | 1.533517 | 1.524882 |
| 436 | 1.42961 | 1.34691 | 1.363549 | 1.290216 |
| 442 | 2.71425 | 0.90524 | 1.301298 | 0.984643 |
| 444 | 1.79365 | 1.12312 | 1.365733 | 1.034862 |
| 450 | 0.9175 | 0.95695 | 1.129504 | 1.137647 |
| 452 | 1.03543 | 1.1482 | 1.433852 | 1.239886 |
| 463 | 0.81652 | 0.64748 | 0.851316 | 0.738094 |
| 467 | 1.35695 | 1.27527 | 1.13442 | 1.33356 |
| 484 | 0.86819 | 0.86181 | 1.037432 | 0.984475 |
| 494 | 0.84813 | 1.1604 | 1.084491 | 1.274495 |
| 497 | 1.06742 | 1.03093 | 0.999803 | 1.118994 |
| 500 | 1.01307 | 1.05564 | 1.231931 | 1.055444 |
| 515 | 1.50829 | 1.64128 | 1.636997 | 1.480288 |
| 517 | 1.37761 | 1.45727 | 1.930919 | 1.479258 |
| 520 | 1.39192 | 1.37821 | 1.853031 | 1.597132 |
| 521 | 1.40462 | 1.51497 | 1.529957 | 1.664871 |
| 522 | 1.35105 | 1.7075 | 1.872204 | 1.523169 |
| 523 | 1.40185 | 1.75371 | 1.833913 | 1.464109 |
| 524 | 1.66272 | 1.69798 | 1.442439 | 1.660106 |
| 526 | 1.8855 | 1.73343 | 1.530556 | 1.654873 |
| 527 | 1.802 | 1.77344 | 1.285924 | 1.554562 |
| 531 | 1.56068 | 1.87842 | 1.905352 | 1.555469 |
| 532 | 1.44599 | 1.82615 | 1.830646 | 1.53189 |
| 533 | 1.91259 | 1.79208 | 1.615161 | 1.77375 |
| 536 | 1.15719 | 1.48578 | 1.3865 | 1.295243 |
| 539 | 1.46897 | 1.6231 | 1.689987 | 1.454022 |
| 541 | 1.77303 | 1.53944 | 1.445971 | 1.489125 |
| 545 | 1.77886 | 1.80683 | 1.836002 | 1.787388 |
| 547 | 1.70061 | 1.75214 | 1.625371 | 1.669106 |
| 548 | 1.6686 | 1.8021 | 1.656037 | 1.694107 |
| 549 | 1.83285 | 1.99911 | 1.670512 | 1.698405 |
| 550 | 1.74384 | 1.9253 | 1.525308 | 1.562826 |
| 551 | 1.45488 | 1.69545 | 1.730623 | 1.55073 |
| 552 | 1.75429 | 1.89889 | 1.66902 | 1.710537 |
| 553 | 1.80318 | 1.82499 | 1.734048 | 1.777233 |
| 554 | 1.81614 | 1.78694 | 1.810063 | 1.637976 |
| 555 | 1.76074 | 1.70281 | 1.729273 | 1.647065 |
| 557 | 1.74646 | 1.76227 | 1.771097 | 1.650033 |
| 559 | 1.57066 | 1.47865 | 1.581973 | 1.525101 |
| 560 | 1.8588 | 1.70375 | 1.826373 | 1.743656 |
| 561 | 2.15861 | 2.03607 | 2.196632 | 2.07154 |
| 562 | 1.97725 | 1.89097 | 1.834899 | 1.691578 |
| 563 | 2.25313 | 2.0314 | 2.174222 | 1.95433 |
| 564 | 4.46908 | 0.88248 | 0.772536 | 0.706088 |
| 565 | 1.36169 | 1.27879 | 1.470883 | 1.85042 |
| 567 | 1.64139 | 1.39553 | 1.315932 | 1.438109 |
| 568 | 1.67367 | 1.26853 | 1.386996 | 1.360807 |
| 573 | 1.41979 | 1.78619 | 1.576017 | 1.873182 |
| 601 | 1.73019 | 1.51188 | 1.860091 | 1.949928 |
| 606 | 0.90209 | 0.94334 | 0.889525 | 1.283893 |
| 608 | 1.49738 | 1.1778 | 1.284249 | 1.269532 |
| 611 | 1.20691 | 0.96681 | 0.963253 | 0.850457 |
| 616 | 1.74366 | 1.17336 | 0.936165 | 1.244311 |
| 660 | 1.61415 | 1.96408 | 1.920275 | 2.123307 |
| 666 | 1.18077 | 1.00375 | 1.199416 | 1.247123 |
| 671 | 0.57409 | 0.6604 | 0.832132 | 0.737345 |
| 694 | 1.08872 | 0.74583 | 0.762928 | 0.91171 |
| 697 | 1.20342 | 1.16721 | 1.099125 | 1.213435 |
| 698 | 0.72238 | 0.58635 | 0.692157 | 0.665094 |
| 699 | 1.06037 | 1.08664 | 1.20415 | 1.165003 |
| 700 | 0.91989 | 1.06605 | 0.998397 | 0.976324 |
| 701 | 0.45131 | 0.50912 | 0.486169 | 0.591247 |
| 702 | 0.59885 | 0.50367 | 0.487489 | 0.613093 |
| 703 | 1.36419 | 1.21216 | 1.199266 | 1.100854 |
| 704 | 2.0361 | 1.31097 | 1.225562 | 1.351382 |
| 706 | 1.29042 | 1.01462 | 0.789415 | 1.13873 |
| 707 | 1.81155 | 1.72582 | 1.502322 | 1.626183 |
| 708 | 1.83465 | 1.78105 | 1.807668 | 1.758726 |
| 709 | 0.83923 | 1.02762 | 1.00238 | 1.054295 |
| 710 | 0.87466 | 0.94464 | 0.969385 | 1.089135 |
| 711 | 0.80575 | 0.86978 | 1.079915 | 0.91275 |
